# International Students’ Mental Health Care in China: A Systematic Review

**DOI:** 10.3390/healthcare9121634

**Published:** 2021-11-25

**Authors:** Yanping Wu, Wuqianhui Liu, Aijun Liu, Li Lin-Schilstra, Ping Lyu

**Affiliations:** 1 School of Overseas Education, Nanjing Xiaozhuang University, 41 Beiwei Road, Nanjing 210017, China; wuyanping@njxzc.edu.cn (Y.W.); lyuping@njxzc.edu.cn (P.L.); 2 The First School of Clinical Medicine, Nanjing Medical University, Nanjing 211166, China; liuwqh@njmu.edu.cn; 3 College of Economics and Management, Nanjing Agricultural University, 1 Weigang, Nanjing 210095, China; li.lin@njau.edu.cn

**Keywords:** systematic review, mental health, international students, China

## Abstract

International students in China are facing difficulties while adapting their Chinese culture, and their life is influenced by the widespread of the Coronavirus Disease 2019 (COVID-19), and caring for their mental health is currently challenging. As a result, our aim is to explore the current mental health care of this minority in China and to provide useful suggestions for future research and institutes. We used the systematic review method, and it was conducted on 11 existing pieces of literature. Our results confirm the unsatisfying psychological situation of international students and the lack of research in this area. We focus on the causes and symptoms of mental problems and explore the effectiveness of methods.

## 1. Introduction

As China’s international education is progressing, this country has been a hotspot for international students, and the number of international students who study in China is constantly increasing. In 2018, there were 492.185 international students from 196 countries in China, among which 59.95% from Asia, 16.57% from Africa, 14.96% from Europe, 7.26% from America and 1.27% from Oceania [1]. International students contribute to the diversity in Chinese Universities and China’s international reputation. Additionally, international students have proven valuable in the study of cultural adaptation and other culture-related studies. However, this minority’s mental situation has not been paid enough attention to. Various factors contribute to the occurrence of mental diseases in international students, including loneliness, isolation and acculturative stress. Particularly, as the widespread of the Coronavirus Disease 2019 (COVID-19) continues to aggravate, quarantine measures have been adopted, and international students’ life in China has been greatly affected during the lockdown. The undeniable impact of COVID-19 on mental health worldwide is causing concern among researchers since the pandemic has led mental diseases, including depression and obsessive-compulsive disorder (OCD) [2], to exacerbate. It is estimated that 13.5% of medical students in China have reported moderate–severe depression even before the pandemic, which indicates an urgent need to handle the problem [3]. International students are vulnerable to various mental diseases, including depression and anxiety [4,5]. Moreover, especially during the pandemic, the Chinese healthcare system is impacted, which brings obstacles to foreigners since many hospitals are shut down or limit the number of patients. International students in this country, however, have received a lack of attention. It is also reported that foreign students in China are suffering from helplessness and increasing anxiety of COVID-19 [6]. As a result, it is indispensable to focus on international students in China because they have to not only suffer from acculturative stress [7] and struggle to adapt to the Chinese culture but also face the great challenges to deal with the epidemic as well. Therefore, we intend to investigate their mental situation and coping strategies based on existing literature during and before the prevalence of COVID-19 and provide suggestions to institutes and clinicians on the prevention of mental diseases of international students in China.

## 2. Method

In this article, we use the systematic review method to summarize the current process of studying the international students’ mental health situation in China. We did not adopt the meta-analysis method for the diversity of mental problems and personal differences among international students in China.

### 2.1. Searching Strategy

We adopt the systematic review method to present studies on the mental health of international students in China. We only included articles that are written in English. Considering the novelty of this theme, we set no time filter, and the earliest article adopted was published in April, 2014. The database review was completed in August, 2021. Pubmed, the Web of Science Core Collection, Medline and Google Scholar are included in the searching process. We choose keywords as follows when proceeding the database research: international student (international student*, foreign student*) in China and mental health (depression, anxiety, disorder*, psycho*). Finally, we found 90 articles. Among them, 86 were found on PubMed, 2 were found on Google Scholar and 2 were found on Web of Science. After removing duplicates, 86 articles were retained. Other study material, for example, the consequences brought by acculturative stress, were collected from PubMed.

### 2.2. Inclusion and Exclusion Criteria

**Figure 1:**
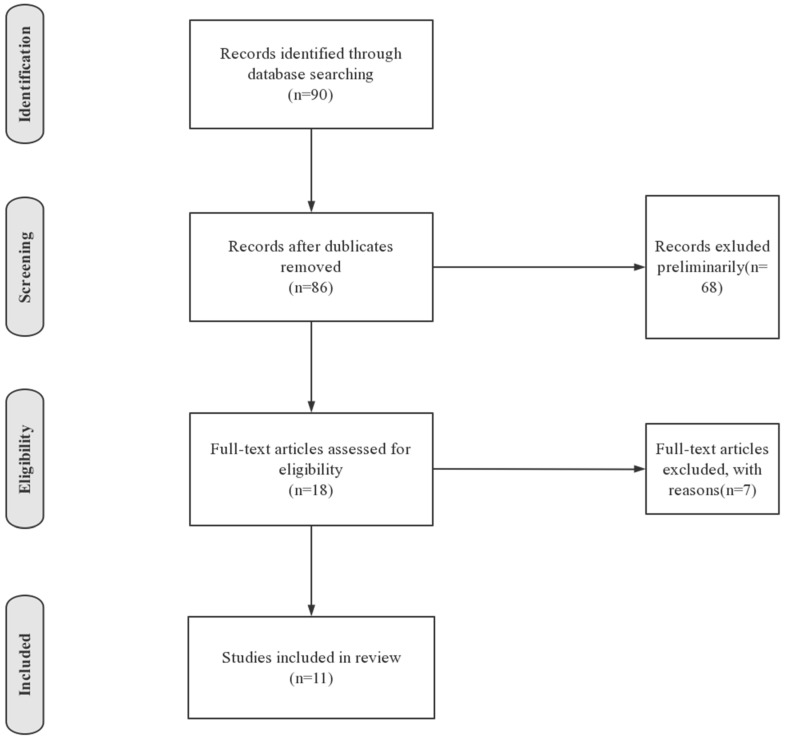
Flow diagram for the process of searching existing literature.

In total, 86 articles were found. Initially, we removes articles that focused on Chinese international students (*n* = 46) and local students in Chinese universities (*n* = 22). Further, articles are excluded if they: (1) explore new methods to conduct education without referring to psychological aspects (*n* = 3); (2) focus on physical problems (*n* = 1); (3) focus on international students in other countries (*n* = 2); (4) are letters to the Editor (*n* = 1). Finally, 11 articles were included. Figure 1 demonstrates the process of including and excluding articles.

## 3. Results

### 3.1. Study of Mental Health Problems and Related Factors

In total, after excluding 4 duplications and 75 unqualified researches, 11 articles were identified [7,8,9,10,11,12,13,14,15,16,17]. Among them, two investigated the learning condition and mental situation of international students in China under COVID-19 [15,17]. One focused on the psychological barriers that influence the efficiency of online education [15]. Four specifically explored depression among various mental health problems [9,12,14,17], and eight referred to depression [7,8,9,10,11,12,14,15]. Loneliness is also a hot issue and is investigated in two articles [8,11]. Two articles chose acculturative stress as their theme [7,12]. All studies are cross-sectional. Table 1 shows the details of all 11 articles. 

Yu et al. [7] examined acculturative stress and its influential factors. Their sample was 567 international students from 97 countries in China, and their data were obtained via the International Student Health and Behavior Survey. They built acculturative stress substructs and analyzed internal and external factors. Their results demonstrated that the acculturative stress level of international students from developing countries in China was about 10 points (M = 92.81 (SD = 23.93) vs. M = 81.39 (SD = 24.66) or M = 83.45 (SD = 25.05)) higher than it of students from developed countries. Students from African (M= 97.66, SD = 23.16) and other Asian (M = 92.54, SD = 22.03) countries reported more acculturative stress than students from countries in other continents (M = 81.19, SD = 27.03). They also determined risk and protective factors for acculturative factors, such as preparedness, religion and marrying status.

Jiang et al. [8] investigated the relation among individualism, loneliness and mobile phone addiction. They conducted an online survey, and their sample was 438 international students from 67 countries. The results revealed that individualism (b = −0.18, *p* < 0.01) had significant and negative influences on loneliness, and loneliness (b = 0.14, *p* < 0.01) had significant and positive influence on smartphone use. Smartphone use (b = 0.17, *p* < 0.01) and loneliness (b = 0.32, *p* < 0.01) showed significant and positive effects on smartphone addiction. Therefore, they came to the conclusion that lower individualism leads to higher loneliness, and loneliness contributes to mobile phone addiction. They also discussed advice and implications for further research.

Liu et al. [9] highlighted the central role of self-confidence in understanding acculturative stress and depression and provided new data supporting more effective counselling for international students in China. They extended the research made by Yu et al. [5]. They conducted a questionnaire data collection among 567 international students in Wuhan, China, and adopted the Acculturative Stress Scale for International Students (ASSIS). Their analysis demonstrated a positive relationship between the total ASSIS score and depression (β = 0.101, *p* < 0.01, F = 143.12, *p* < 0.001). Their results also indicated that low cultural competence, homesickness and low self-confidence were significantly and positively associated with depression, and they emphasized the importance of self-confidence in the role of preventing depression.

Ansong et al. [10] addressed menstrual problems, together with their associated risk factors, among international students in China. This cross-sectional study included 2016 female international students in China. They found out high stress was also significantly associated the risk of dysmenorrhea (*p* = 0.037). This study also indicated that over half (60.4%) of the studied population suffer from high stress. As a result, it is necessary to provide support according to other studies, including religion, mental health and cultural background. Their conclusion was that high level stress leads to menstrual disorders, and menstrual disorders rate is high in international students in China.

Jiang et al. [11] aimed to explore life satisfaction of international students in China. This article is a comprehensive review for policies and practice and other reviews from various aspects related to international students in China, along with studies in other countries. They discussed present polices, political factors and the situation of international students. As for psychological aspects, they indicated that a minority of students have mental problems, and many are suffering from loneliness and sadness.

Shan et al. [12] investigated acculturation stress among Pakistani students in China. Their sample was 203 Pakistani university students in China. The ASSIS was adopted. They estimated the degree of acculturative stress among Pakistani students, along with discrimination, homesickness, stress and fear. They finally offered useful suggestions for the improvement of their mental health.

Hu et al. [13] used the Cognitive Emotion Regulation Questionnaire (CERQ), College Students’ Life Events Scale (ASELC) and SDS to investigate depression symptoms among university students, both local and international, in China. They conducted a comparison between international and local students between male and female ones. They suggested that compared to Chinese students, international students more often used cognitive adjustment methods. Their conclusion was that positive coping methods lead to the declination of depression and vice versa.

Wang et al. [14] conducted an online survey on the mental health status under COVID-19 of international students in Changsha, China. Their study demonstrated that the prevalence of depression was 59.4%, and the prevalence of anxiety was 37.8%. As for students under quarantine, researchers did not find rise in the rate of anxiety and depression. The study suggests that schools need to consider providing short-term and long-term psychological help services for international students.

Li et al. [15] studied the effect of social support on depression and mediation mechanisms in international students. Their study was conducted on 349 international students and their depression extent was evaluated by the SDS and Social Support Rating Scale. The results showed that social support had a significant predictive effect on attachment closeness (β = 0.110, *p* < 0.001), and the interaction between social support and self-esteem had a significant effect on attachment closeness (β = 0.020, *p* < 0.05). The effect of social support on depression was significant (β = −0.325, *p* < 0.001), and the effect of attachment closeness on depression was also significant (β = −0.305, *p* < 0.05). Their conclusion was that in the relationship between social support and depression, self-esteem plays a moderating role in the mediating effect of attachment closeness.

### 3.2. Studies of Psychological Measures That Affect Students’ Mental Health

Gu et al. [16] investigated the effects of mindfulness training on depressive symptoms of international students. They adopted the Self-Rating Depression Scale to estimate the depression rate of students. Their results suggest that 33.80% of their sample have mild depression, 17.84% of their sample have moderate depression and 5.16% have severe depression. The mean depression degree of male students and female students was 49.93% and 42.55%. The mean depression index for all participants was 0.46, which indicated a mild depression. Having analyzed their depression rate after receiving mindfulness training, they came to the conclusion that mindfulness training and positive coping style are interrelated with treating depressive symptoms for international students. 

### 3.3. Studies of Other Measures Adopted during COVID-19 Affecting Students’ Mental Health

Li et al. [17] studied the quality of online education under COVID-19. This study pointed out several crucial factors that influence students’ satisfaction of online education. They indicated that international students who are far away from home are vulnerable to mental health problems and advised that schools provide support for international students who stayed in China during the pandemic. Their study mainly focused on both internal and external factors that influence the quality of online education during the quarantine. 

## 4. Discussion

### 4.1. Mental Health Situation of International Students

#### 4.1.1. Depression and Anxiety Symptoms

We can conclude from the literature included that international students in China have a relatively high rate of depression compared to normal Chinese people, whose depression rate is 2.1% [18]. According to Wang et al. [14], the prevalence of depression among international students is 59.7%, and according to Gu et al. [16], the prevalence of it is 56.8%. Those two values are close to each other, increasing the reliability of the results. These suggest an approximately 27 times higher prevalence than ordinary Chinese residents, indicating a serious mental health crisis. Afterwards, China’s international students’ anxiety level is higher than that in South Korea (49%) [19]. Acculturative stress, high academical pressure and loneliness probably account for this phenomenon. As can be seen from the results, among all 11 studies, eight only focus on depression and anxiety symptoms, which cover 72.7% of the studies, and other mental diseases are not involved in researches. However, it is notable that over 2% of the whole population around the world is suffering from OCD [2], and other severe mental problems, such as schizophrenia and bipolar symptoms, that occupy a certain part of the population. The sole focus on depression and anxiety is a one-sided issue of the picture. Anxiety, OCD and depression have common points and can all be treated with Selective Serotonin Reuptake Inhibitors (SSRIs) [20], suggesting the need of investigating various mental problems, which may also provide a chance to find out the relationship between different mental health problems. Moreover, studies on multiple mental problems may provide more angles to solve students’ difficulty and to explore the interaction between simple neurosis and complicated mental problems.

#### 4.1.2. Acculturative Stress

Acculturative stress is defined as the process of confronting challenges in cross-cultural exchange settings [7], which is one of the multiple adaptation problems for international students [21]. This stress leads to various problems, including alcohol addiction, emotional eating and sleeping obstruction [22,23,24]. International students are ideal samples to investigate this issue, which is even more obvious in China since this country has students from both developed countries and developing countries. Two studies in this review ([7,12]) solely focus on this unique problem. Better preparedness is beneficial for the solution of acculturative stress [7,12]. As a result, universities can provide useful lectures about Chinese culture before international students arrive in China or in the orientation courses to make them more well-prepared and to decrease the effect of acculturative stress. 

#### 4.1.3. Variation in Mental Health Situation under COVID-19

China mainly adopts quarantine measures in order to control the spread of COVID-19, which have proven to be effective [25]. During the lockdown, about 75% of international students came back to their countries [17]. Nonetheless, international students’ life are greatly affected under the strict quarantine measures. According to Wang et al. [14], some international students are quarantined in their dormitories, and the length of quarantine time affects the possibility of having intellectual problems. According to Li et al. [17], other international students staying in their own countries are provided with online courses. As a result, they may feel isolated, and their study is not as satisfying as before, and the exacerbation of loneliness occurs [17]. Thus, universities should manage to provide a better learning environment for students staying abroad and, at the same time, offer effective psychological services for all international students. Moreover, further studies are expected on the comparison of the anxiety level of students, both local and foreign, before and after vaccination.

### 4.2. Coping Strategies and Implication for Universities, Researchers and Governments

We can summarize from the analysis presented above that international students’ mental health situation is not satisfying, so it is urgent to do more research and improve coping strategies. Two studies [9,16] emphasized the importance of self-esteem and self-confidence in the prevention of depression, which is also supported by [26,27]. Teachers may consider giving courses that give rise to an increase in self-confidence. Gu et al. [16] confirmed the usefulness of mindfulness training since it leads to the declination of depression symptoms, which is also supported by McConveille et al. [28]. However, because this is the sole research that provides the evidence of the benefit of adopting psychological measures, it can be deduced that few universities in China conduct psychological courses for international students. Therefore, it is high time that universities improve related services for international students. Psychological teachers’ English level should be approved, making it available for international students to counsel. The effect of counseling between Chinese teachers and foreign students should also be tested by other researches. There are totally 11 qualified articles, indicating a need for more research. Research on other meditating methods will be helpful. Additionally, a cross-country comparison is recommended. It can be seen from the results that all studies are cross-sectional. If teachers and clinicians are able to use panel data to investigate students’ psychological problems, new angles may be provided. 

As for the Chinese government, better policies should be introduced. International students who stay in the mainland may enjoy a discount when buying psychotropic drugs, or the cost may be included in their medical insurance. Moreover, a great many hospitals in China nowadays have international medical centers that provide special services for foreigners, and some hospitals include psychological counseling in these centers. These institutions can be introduced to more hospitals and may cooperate with international centers in colleges. They may promote their services to foreign students at a reasonable price. 

## 5. Conclusions

The mental health care of international students in China is not satisfying, and the attention paid to this minority is insufficient. Under COVID-19, variation occurs in their life, and they need to adapt. As can be seen from the results, there is little research in this area. Despite abundant research of depression and anxiety, other diseases are not included. Researches in each aspect are not comprehensive enough. Chinese universities should provide better services for international students. The cooperation between the Chinese government and universities is indispensable for the improvement of international students’ mental health. Further research is needed on the interaction among different mental diseases and on more effective measures to improve their mental health. There is still a lot of room for this issue to be improved.

## Figures and Tables

**Table 1:** Data extraction of included studies.

Author and Year	Purpose	Data Sources	Study Design/Sample Size	Control Variables	Measures	Findings
[5] Yu et al., 2014	To investigate acculturative stress among international students in China and the influential factors behind it	questionnaire data	Cross-sectional/*n* = 567	country, age, religion, months in China, marital status	Acculturative Stress Subconstructs	Acculturative stress is more common in China than developed countries and in students that are unmarried and well-prepared and do not belong to an organized religion.
[6] Jiang et al. 2018	To examine the relationship between individualism, loneliness, smartphone use and smartphone addiction	questionnaire data	Cross-sectional/*n* = 438	country, age	Hofsted’s Individualism Index, 32; the UCLA Loneliness Scale, 26.67 Smartphone Addiction Scale–Short version (SAS-SV).50	International students with lower degrees of individualism show higher degrees of loneliness, which leads to a higher degree of smartphone use and smartphone addiction.
[7] Liu et al., 2016	To investigate the relationship between various components and depression	questionnaire data	Cross-sectional/*n* = 567	sex, age, country	Acculturative Stress Scale for International Students; Center for Epidemiologic Studies Short Depression Scale	The role of self-confidence in understanding is crucial in acculturative stress and depression.
[8] Ansong et al. 2019	To address the menstrual problems, together with their associated risk factors, among international students in China.	questionnaire data	Cross-sectional/*n* = 409	age, BMI, country, program	N/A	A high level of stress leads to menstrual disorders, and menstrual disorders rate is high in international students in China.
[9] Jiang et al., 2020	To explore life satisfaction of international students in China	existing literature	N/A	N/A	N/A	Findings are helpful for further research, policy makers and university practitioners to provide high quality education.
[10] Shan et al., 2020	To investigate acculturation stress among Pakistani students in China	questionnaire data	Cross-sectional/*n* = 203	N/A	N/A	Orientation lectures, interaction with local students and related activities are helpful for international students.
[11] Hu et al., 2016	To investigate the CERQ results and depressive symptoms of both local and international students	questionnaire data	Cross-sectional/*n* = 255(foreign students), *n* = 262 (Chinese students)	age, sex	CERQ; ASLEC; SDS	Encouraging students to use adaptive coping methods during psychological intervention is an effective way to adjust cognitions and behavior.
[12] Wang et al., 2020	To investigate the mental health status of international students from Changsha city, China	questionnaire data	Cross-sectional/*n* = 153	school time, sex, age	PHQ-9; GAD-7; SATI	The study implies that the university needs to consider planning for acute and long-term psychological help services for international students.
[13] Li et al., 2021	To investigate the influence of social support on depression and the mediation and moderation mechanisms among international students	questionnaire data	Cross-sectional/*n* = 349	N/A	Self-Rating Depression Scale; Social Support Rating Scale	Attachment closeness has an effect on depression; the direct effect of social support and the mediating effect of attachment and closeness are regulated by self-esteem.
[14] Gu et al., 2020	To investigate the effects of mindfulness training on depressive symptoms of international students	questionnaire data	Cross-sectional and panel/*n* = 260	country, sex, major	Self-Rating Depression Scale; Life Event Test	Mindfulness training for 8 weeks significantly reduced the depressive symptoms.
[15] Li et al., 2021	To explore the quality of the online education in China for international medical and nursing students from developing countries	questionnaire data	Cross-sectional/*n* = 230 (student), *n* = 95 (teacher)	major, age, months in China, sex	N/A	The study defines several factors that affect the quality of online education for international students.

SATI: State-Trait Anxiety Inventory; GAD-7: 7-item Generalized Anxiety Disorder-7 Scale H; PHQ-9: the 9-item Patient Health Questionnaire-9; CERQ: The Cognitive Emotion Regulation Questionnaire; SDS: A Self-rating Depression Scale; ASLEC: College Students’ Life Events Scale.

## Data Availability

No data, models, or code were generated or used during the study.
